# Job Search during COVID-19: How Online Mindfulness Intervention helped to Reduce Stress and Enhance the Self-esteem of the Job Seeker

**DOI:** 10.12688/f1000research.109523.2

**Published:** 2022-11-04

**Authors:** Rajalakshmi S.A., Sowndaram C.S., Preetham Ganesh, Harsha Vardhini Vasu

**Affiliations:** 1 Department of Language and Humanities, Amrita School of Engineering, Amrita Vishwa Vidhyapeetham, Coimbatore, Tamilnadu, 641112, India; 2 Department of Computer Science and Engineering, Amrita School of Engineering, Amrita Vishwa Vidhyapeetham, Coimbatore, Tamilnadu, 641112, India

**Keywords:** COVID-19, placements, college students, mindfulness, stress, self-esteem

## Abstract

**Background: **Immediately after graduation from university, college students need to make significant decisions about starting their careers or pursuing higher studies. They are also pressured to meet the expectations and demands of self, others, and the environment. Owing to the impact of the COVID-19 pandemic, the aforementioned challenging decisions may become hazardous stressors for college students. Hence, the researchers intended to assist and assess the college students involved in student placements. The research goal was to investigate the impact of mindfulness-based intervention (MBI) on the stress and self-esteem of college students involved in student placements.

**Methods: **One hundred college students participating in the campus placements were selected using purposive sampling from Amrita Vishwa Vidyapeetham University in Coimbatore, India. For evaluation purposes, college students were administered the perceived stress, Rosenberg self-esteem, and Kuppuswamy socio-economic scales. Seventy-five college students were selected for the MBI process and were administered with a pre-intervention and post-intervention without a control group research design.

**Results: **Statistical analysis including analysis of variance (ANOVA) and the Bonferroni post hoc test showed a significant increase in self-esteem and a decrease in the stress of the college students involved in placements.

**Conclusions: **Thus, the researchers recommend that policymakers create awareness, include MBI in the curriculum, and allocate funds for training ventures in educational institutions to assist college students in their challenging life journeys

## Introduction

Unemployment is one of the critical problems many people face across the globe. Some of them are new graduates, employees who have been laid off, and individuals who try for better options than their current employment. Students can find employment either by attending interviews with companies or by attending job fairs. Labour market opportunities are open to individuals with higher degrees and more experience ^1^. Indeed, the global pandemic COVID-19 has impacted all the spheres of human lives, and the job market has also been affected. European countries were significantly impacted due to the COVID-19 pandemic. The countries had imposed multiple lockdowns, due to a high number of cases and deaths, which led to economic decline, and resulted in companies reducing their budget for hiring new employees ^2^. During COVID-19, in Sweden, job seekers did not show much interest in applying for high profile careers, which reduced the new job announcements from the recruiter’s side ^3^. India’s urban and rural areas suffered because of COVID cases, lockdown, and economic decline, and all these issues contributed to the rise in unemployment ^4^.

In general, students who try to find a placement in established corporate companies have high stress and low self-esteem as they do not have pre-exposure to corporate companies ^5^. Research on Israel’s younger population showed a connection between unemployment, COVID-19, and mental distress. This mental distress increased due to financial insecurity and aloofness ^6^.

Stress takes time to build and does not come overnight. Likewise, self-esteem lowers gradually due to a sequence of events and not just one event in an individual’s life. Stress can be of two types: acute and chronic ^7^. The chronic stress caused by unemployment may lead to low self-esteem, and with the help of mindfulness training it can be handled well ^8^. Factors like gender and college students’ perception of parenting style can also impact their self-esteem ^9^. Introverted college students showed more association with low self-esteem ^10^.

Before the COVID-19 pandemic outbreak, institutions opted for placement coaching to solve placement-related mental health issues. In addition, there are choices for stress reduction like yoga meditation ^11^, self-reiki ^12^, progressive muscle relaxation ^13^, and many more. Likewise, self-esteem may improve in several ways, such as self-uncertainty salience ^14^, Yoga Nidra ^15^, materialism ^16^. The process of bringing awareness of the outer and inner occurrences in the current moment is called mindfulness ^17^.

Before everything else, the impact of the pandemic on college students needs special consideration. As an after-effect of the pandemic, college students may experience anxiety and depression due to a decline in family income, loss of housing, infection with the coronavirus; hence, they require a psychological support system. Ultimately, college students have to be handled with a method that addresses multidimensional issues ^18^. College students were isolated inside their homes because of COVID-19, which caused severe mental health issues due to problems such as separation from other people, increased psychiatric symptoms, and non-availability of the necessary information related to the pandemic ^19^. Owing to the pandemic, college students’ took less time for important tasks such as sleeping, eating, and communicating with their peers. They also started spending more time using electronic gadgets. Severance of one’s routine results in depression and other mental health issues ^20^. The adverse effects of COVID-19 made some college students endure psychological distress and other symptoms. Despite these issues, they were able to adapt and learn new technology and survive amidst the crisis ^21^.

Mindfulness-based intervention is a gentle method of treatment that can harmonize the mind and the body. Mindfulness techniques have been researched by many, and some of these methods include: using the functional near-infrared spectroscopy to identify the dissimilarity of the hemodynamics of participants ^22^; examining enhancements of brain indexes of consciousness process using event-related potentials after giving school-based mindfulness ^23^; using voxel-based morphometry to identify the neural relationship of solitary differences in trait mindfulness ^16^; using electroencephalogram and electrocardiogram to record the brain and heart activities and study the relationship between the signals during their reaction to mindfulness-based stress reduction ^24^; using mindfulness on stroke and motor neuron disease patients and comparing the performance with music training while using electroencephalogram-based brain-computer interface devices ^25^, and many more.

Mindfulness has various applications among students, but the needs of college students are different to school students. Researchers have performed mindfulness-based studies with college students in many ways, some of which are listed as follows: mindfulness has been applied to improve self-compassion and regulation of emotion in college students by using yoga-based meditation techniques ^26^; examining the influence of protective behavioural strategies as a mediator on the impact of mindfulness on repercussions related to effects of alcohol consumption ^27^; evaluating the role of therapeutic groups like mindfulness, relaxation, and control on outputs like drinking urge, negative and positive effects among students prone to alcohol consumption ^28^; assessing the effects of mindfulness and behavioural activation on a waitlist control group ^29^.

However, mindfulness studies conducted with school students focused on different aspects. It has been applied to increase the focus of school students and it resulted in a positive outcome ^30^. For example, digital game-based creativity learning has been used to identify the relationship between goal, self-determination, mastery experience, and mindfulness ^31^. The influence of mindfulness has also been used to estimate the creativity level in graphics in a group of Latin American teenagers ^32^, decreasing illness linked with corpulence and psychology disorders ^33^.

Studies indicate that mindfulness has extreme benefits with problems such as chronic disease ^34^ including heart disease ^35^, bowel disease ^36^, lung disease ^37^, insomnia ^38^ and leukaemia ^39^.

College students who received online mindfulness training showed a reduction in anxiety and depression levels caused by COVID-19 ^40^. The repercussions of the COVID-19 pandemic impacted both teachers and students. Students showed a decline in their learning performance but mindfulness practices could address these issues ^41^.

Although the COVID-19 pandemic did not offer us many options, the efficacy of online mindfulness interventions raised concerns among practitioners. Mindfulness intervention was offered through a smartphone application, Headspace ^42^ to employers, and the results showed that consistent practice aided in the reduction of stress and enhancement of psychological well-being ^43^. The competence of mindfulness training in a video-based streaming service named Spectiv was found to facilitate mindfulness and well-being effectively ^44^. Mindfulness-based interventions may assist the student placement process and the impact caused by COVID-19. Kam *et al*. ^45^ found that even brief 10-minute mindfulness meditation practice on a daily basis will mitigate the negative effect caused by the constant exposure to COVID-19 news. Researchers applied mindfulness-based interventions to college students involved in placement earlier, but this study is unique because of the pandemic situation and its complexities.

In this experimental study, we analyzed the impact of mindfulness-based interventions on college students involved in placement training. We hypothesised that mindfulness-based interventions would reduce stress and enhance self-esteem that both the placement and COVID-19 would likely impact. Therefore, we assume that consistent mindfulness meditation practices by college students will bring positive changes in stress, self-esteem, and the after-effects of the COVID-19 pandemic.

### Objectives

- To assess the level of self-esteem among college students involved in a placement.
- To evaluate the stress levels among college students involved in a placement.
- To estimate the effect of mindfulness-based interventions among the college students involved in a placement.
- To assess the significant difference between mindfulness-based interventions in the pre, post, and follow-up phases of the study.

### Hypotheses

Our hypotheses in the following study were:

- There will be a significant change in the self-esteem of the college students involved in the placements during the COVID-19 pandemic pre, post and follow-up phases of mindfulness-based interventions.
- There will be a significant change in the stress of the college students involved in the placements during the COVID-19 pandemic pre, post and follow-up phases of mindfulness-based interventions.
- Mindfulness-based interventions will enhance self-esteem and mitigate stress among college students involved in the placements during the COVID-19 pandemic pre, post and follow-up phases.

## Methods

### Study design

This research follows a quasi-experimental design. The researchers initiated a focused group discussion among the researchers, teachers, and counselors to discuss the methods and procedures required. The members approved purposive sampling, online data collection, and intervention owing to the COVID-19 and convenience factors. Although members suggested different questionnaires, the unanimous consensus was to use the perceived stress scale, Rosenberg’s self-esteem scale, and the Kuppuswamy socio-economic scale. The researchers decided on questionnaires after carefully validating the objectives and suitability of the questionnaires to the Indian culture. The discussions led to checking the reliability and validity of the questionnaires to ensure the quality of the research. The members also suggested a pilot study to confirm the proposed intervention plan.

### Setting

The focused group members are all from the Amrita School of Engineering, Coimbatore and devised the research strategy from first-hand experience with the potential participants. Thus immediately after the focused group discussion, researchers framed the research outline and sought ethical clearance.

### Procedure

**Figure 1.**
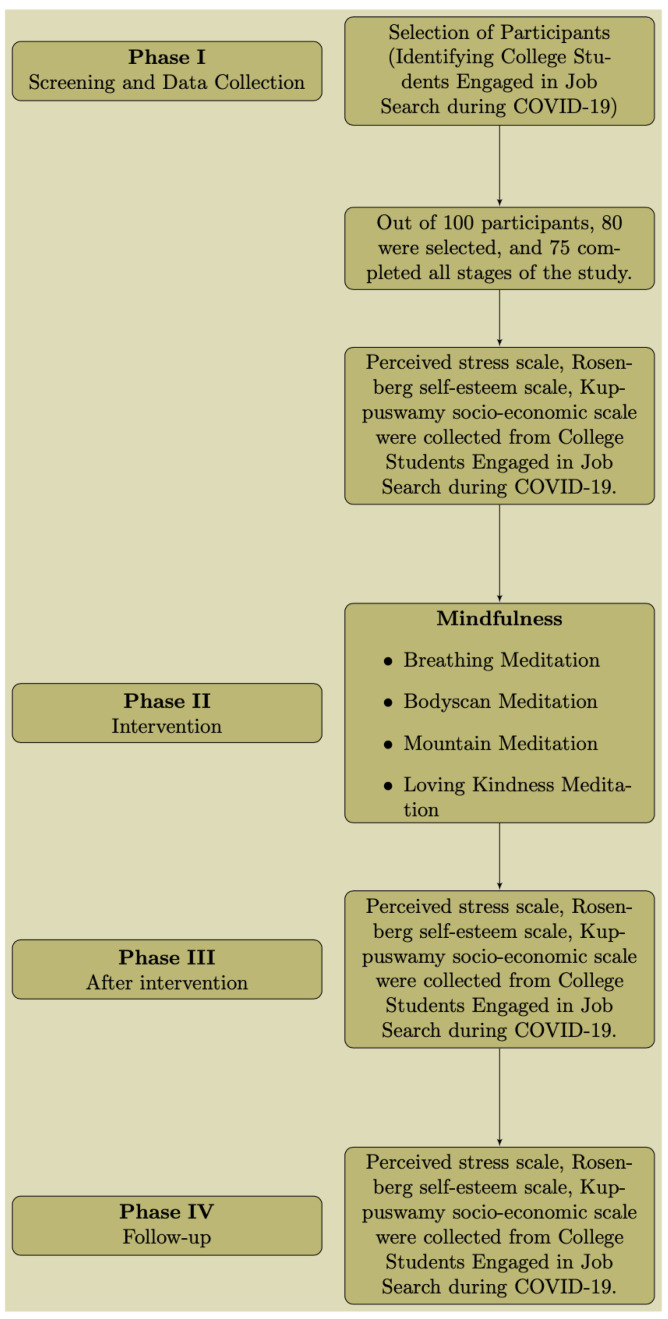
Process flow.

This research gained approval and ethical clearance (AMRITA/SOE/ADMN/DOE/01/2021/01) from the Ethics Committee, Amrita School of Engineering, Amrita Vishwa Vidhyapeetham, Coimbatore, India. Written informed consent was obtained from the participants on the Google form. Participants were also given the necessary details about the intervention outcomes and assured that they had full rights to withdraw at any point of the research. The data collection and analysis were done based on the ethics committee, Amrita Vishwa Vidhyapeetham, India, and the personal identification details of the participants were removed. The tools (Perceived stress scale, Rosenberg self-esteem scale & Kuppuswamy socio-economic scale) ^46, 47^ were added to a Google Form, and a total of 100 students completed the form. Then every student’s stress and self-esteem were calculated. Although the Google form had data from 100 students, not all had high stress and low self-esteem. Hence, a threshold value was chosen for both the variables, where the value for stress was 22 and self-esteem was 31. A total of eighty students fell into this category, upon which they were sent emails regarding their scores and the option to attend the mindfulness-based intervention. Out of the 80 students, 75 responded positively and attended the intervention. Figure 1 indicates the process flow of the study.

The stress and self-esteem scores before the intervention were taken from the Google form, and after the intervention, the students were asked to fill out a new Google form containing the same questionnaires. A follow-up was conducted using the same set of procedures one month after the intervention. The results were analyzed using SPSS 23.0 (IBM SPSS Statistics: RRID: SCR_016479) ^48^.

### Participants

Purposive sampling was used to select college students involved in the placement process at Amrita Vishwa Vidyapeetham University in Coimbatore, India. The inclusion criteria were: (1) Students with high stress and low self-esteem or either of the above. (2) Students who were in their final year of college, preparing for their placements. (3) Students with a grade point average of more than 6.5 out of 10 (University placement eligibility policy). The exclusion criteria were: (1) Students who were preparing to pursue higher studies inside or outside India. (2) Students who were planning start-ups. One hundred students who fit the criteria mentioned above, completed a demographic survey shared with them through Google Forms.

### Variables

In this research, self-esteem is the perception of the sense of worth of the college student involved in placement possess. This self-esteem may have varied due to the adverse situations produced by placements during COVID-19. Stress is the perception of burden and negative feeling due to a problem, person or thing caused by the self-evaluation that they lack the specific skills required to handle that situation. This stress may lead to physical disturbances also. However, physical disturbances were not included in the scope of the study. Henceforth, stress was defined as an overtaxing, and heavy feeling and thinking in college students caused by the placements during COVID-19.

### Data source/measurement

A demographic survey was used to collect participants’ data such as name, age, class, gender, and consent to participate in the intervention. The students were informed about confidentiality and that their details would not be revealed to anyone under any circumstances. At the end of the intervention, the students were asked to provide feedback (if any). The following tools were used to collect data:

### Perceived stress scale

The perceived stress scale (PSS) was designed by Cohen *et al*. ^49, 50^ to measure the perceived stress in individuals. It is a five-point Likert scale ranging from zero to four with the options of never to not very often. It is a 10 item instrument where the total score is summed, and the higher the score, the higher the perceived stress. Some of the items are, "In the last month, how often have you been upset because of something that happened unexpectedly?", "In the last month, how often have you felt that you were on top of things?". This scale’s reliability was assessed by pre-testing it to one hundred and six college students participating in campus placements. The Cronbach’s alpha value of 10-item PSS indicates moderate internal consistency of 0.79, and test-retest reliability for a week showed, Intra-class correlation (ICC) of 0.83. During focused group discussion, the expert team authorized the face validity and content validity of the same.

### Rosenberg self-esteem scale

The Rosenberg self-esteem scale is a 10 item scale for estimating the value of an individual’s self-esteem, it has a four-point Likert scale ranging from strongly agree to disagree strongly ^50, 51^. Some of the statements are: "I certainly feel useless at times" and "I take a positive attitude towards myself". While conducting the focused group discussion, face validity and content validity were discussed: a pre-testing of this 10-item scale was carried out on 106 college students involved in campus placements. Results showed that Cronbach’s value for internal consistency was 0.85 and ICC value for test-retest reliability was 0.86.

### Kuppuswamy socio-economic scale

The Kuppuswamy’s socio-economic scale ^50, 52^ is mainly used to measure the status of a person in society by considering parameters such as education, occupation, and salary earned by the head of the family. The parameters, as mentioned earlier, have further subgroups where each has individual scores, the total of which ranges from 3–29. Based on the total score, the students were grouped into the following five classes: upper class, upper-middle class, lower-middle class, upper-lower class, and lower class.

### Mindfulness-based intervention

The mindfulness-based intervention used in this research was inspired by Kabat-Zinn ^17, 50^. The intervention used in this experiment combined five mindfulness meditation techniques and mindfulness-based activities. The outline of the interventions used is tabulated in Table 1. Eating meditation helps to focus on the raisin's texture, colour, odour, and flavour and savour it in slow and steady movements by enjoying each observation process. Breathing meditation brings the consciousness to breathing and feeling in tune with inhaling and exhaling than other distractions. Body scan meditation is scanning the body parts from head to foot aligned with breathing and letting the stress and anxiety move out of the body. Mountain meditation slowly brings the mountain into one's consciousness and becomes a mountain itself. By doing this, one can become a confident and stoic person facing all the happenings with unchanged vigour. Loving-kindness meditation sends love and kindness to self, known, strangers, enemies, and all living beings in a deeply relaxed state. The focus is on forgiving and compassion for all, irrespective of past and present happenings. All these mindfulness meditations helped college students to relax and have enhanced self-esteem.

**Table 1.** Mindfulness-based interventions outline.

Week	Contents	Outcome
1	Introduction about the program	College students understood outline of interventions
	- Definition of terminologies	Experienced eating meditation.
	- Outline of the planned activities	
	- Utility factors and Eating meditation	
2	Body scan and breathing meditation.	Students experienced body scan & breathing meditation.
3	Introduction to Mountain Meditation	Students gained flavor of mountain meditation
	- Uses and its connection with the brain.	- Understood the importance of practicing.
4	Mountain Meditation (practice session).	Students strengthened connections with meditation.
		- Clarified their practical difficulties associated with it
5	Introduction to Loving-Kindness Meditation	Students experienced & appreciated the benefits of it.
6	Loving-Kindness Meditation (practice session)	Students experienced a deeper connection with practice.
7	- Reflection of self	- Students had space to reflect on intervention process.
	- Revisiting of past sessions	- Students also spoke about connection with themselves.
	- Explanation of connection between full process	- Students expressed views on practising meditations.
	- Group discussion and feedback	- Students also gave feedback on the intervention.

The sessions were conducted online using Google meet, and each session was for two hours. Thus, the students attended one session weekly and, overall, eight sessions. In addition, the college students were asked to practice at least 30 minutes of mindfulness meditation on their own and report to the researchers. The college students were eager to oblige, and they completed the practice for eight weeks and reported their progress weekly once a month.

### Statistical methods

The quantitative measures were analyzed using SPSS 23.0, segregating the research process into pre, post, and follow-up phases. The demographic variables, namely age, gender and socio-economic status, were analyzed using percentage analysis. Researchers calculated the mean and standard deviation to understand the changes in the variables during pre, post and follow-up phases. One-way ANOVA portrayed the significant difference caused in the variables because of mindfulness-based intervention. Post hoc analysis was employed to identify the significant changes during the pre, post and follow-up phases of mindfulness-based interventions.

### Preliminary pilot testing

The researchers conducted a pilot study to ensure the feasibility of the design and outcome. Out of fifty involved in the placements, thirty had high stress and low self-esteem and among them, a few dropped out due to personal and academic difficulties. The pilot study started by pre-testing the twenty-one college students with the Perceived stress scale and the Rosenberg self-esteem scale followed by a mindfulness-based intervention: eating meditation, breathing meditation and body scan meditation for two weeks (two sessions, total four hours). It was found that the pre-phase mean value of stress was 28.05, and the post was 24.33. Similarly, the pre-phase self-esteem value was 23.80, and the post was 25.85. A one-way ANOVA test showed that there was a significant difference in the stress scores during the pre- and post phases of the mindfulness-based intervention (F (1,40)) = 11.69, p = 0.00). However, for self-esteem the one-way ANOVA indicated that there was no statistically significant difference between the pre- and post phases (F (1,40)) = 2.47, p = 0.12). Although there was a rise in the mean score, it was not statistically significant. In the focused group, discussion members suggested the following inclusions and those were carried out in the study. Expanding the intervention time from two weeks to eight weeks and including the meditation methods such as mountain meditation and loving-kindness meditation A session was devoted solely to connecting with meditation, self-reflection on its benefits and committing to understand and practice MBI.

## Results

### Participants

Although 80 students needed the mindfulness-based intervention, five members opted out due to conflict with college practical lab sessions and personal reasons. Thus, the mindfulness-based intervention commenced with 75 members, and all of them attended all three phases: pre, post and follow-up.

Table 2 shows the demographic variables of the college students. The college students involved in the study were from 19 to 21 years of age, of which 48% of the population were 20-years-old, while 12% were 21-years-old. Twentynine percent of the students were female, and 71% were male. The absence of the socio-economic classes upper-lower and lower among the college students was noted. The majority of the college students were from upper-middle class (50.67%) and upper class (46.67%) backgrounds, while a few were lower-middle class (0.02%). 

**Table 2.** Demographic details of the college students (N=75).

Demographic variables	Subgroups	Number of participants (%)
Age	19	30 (40%)
	20	36 (48%)
	21	9 (12%)
Gender	Male	53 (71%)
	Female	22 (29%)
Socio-economic status	Upper	35 (46.67%)
	Upper-middle	38 (50.67%)
	Lower-middle	2 (0.02%)
	Upper-lower	0
	Lower	0

Table 3, shows the pre-phase mean value of stress was 22.57, which is higher than the post-phase mean value 18.25, indicating that the intervention has reduced the stress levels in the participants. Similarly, the pre-phase mean value of self-esteem was 25.92, which is lower than the post-phase mean value 28.90, indicating that the intervention increased self-esteem among the participants. During the follow-up phase, the mean values for stress and self-esteem were 18.04 and 29.09, respectively, and these scores show that the impact of the intervention was sustained.

**Table 3.** Collective measures of stress and self-esteem for the participants during pre-, post-, and follow-up phases.

Variables	Pre-		Post-		Follow-up	
	Mean	S. D	Mean	S. D	Mean	S. D
Stress	22.57	4.28	18.25	4.79	18.04	4.70
Self-esteem	25.92	3.56	28.90	3.68	29.09	3.61

N = 75 S. D: Standard Deviation

Table 4 shows the approximate F calculation of the pre-, post-, and follow-up phases for both the variables (self-esteem and stress). The output of analysis shows that for self-esteem, there was a statistically significant difference between pre-, post-, and follow-up phases as determined by one-way ANOVA, (F (2,222)) = 23.21, p = 0.00). There was also a statistically significant difference between the pre-, post- and follow-up phases for stress as determined by one-way ANOVA (F (2,222)) = 18.15, p = 0.00). The results indicated that mindfulness-based interventions helped college students during the placements.

**Table 4.** Approximate F for the pre- and post-phases of stress and self-esteem.

Variables	Sum of squares	Df	Mean square	F
Stress	475.62	2	237.81	18.15 **
	2908.21	222	13.10	
	3383.84	224		
Self-esteem	981.47	2	490.73	23.21 **
	4693.41	222	21.14	
	5674.88	224		

** Significance at 0.01 level Df: Degree of Freedom

**Figure 2.**
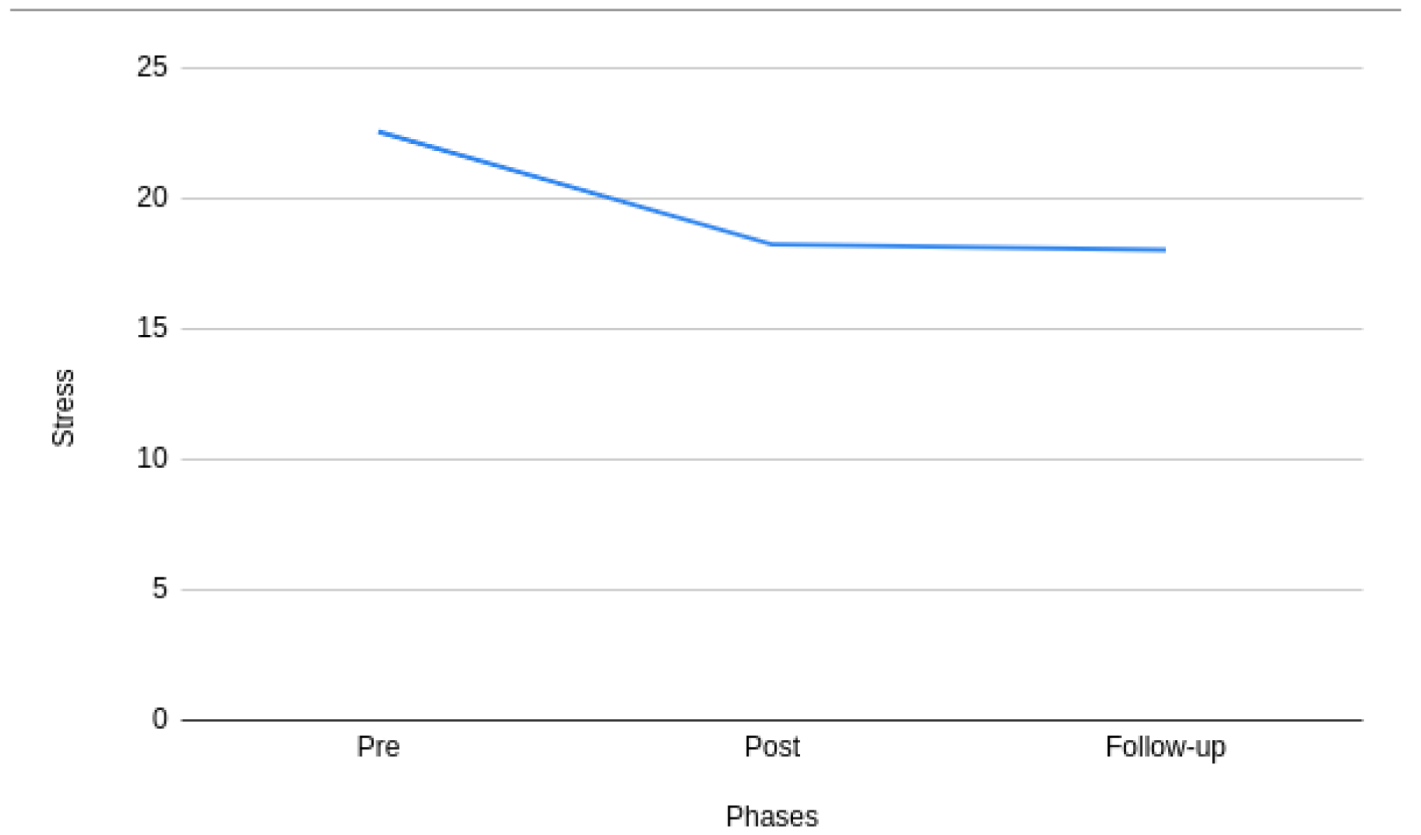
Pre-, post-, and follow-up phases of stress.

**Figure 3.**
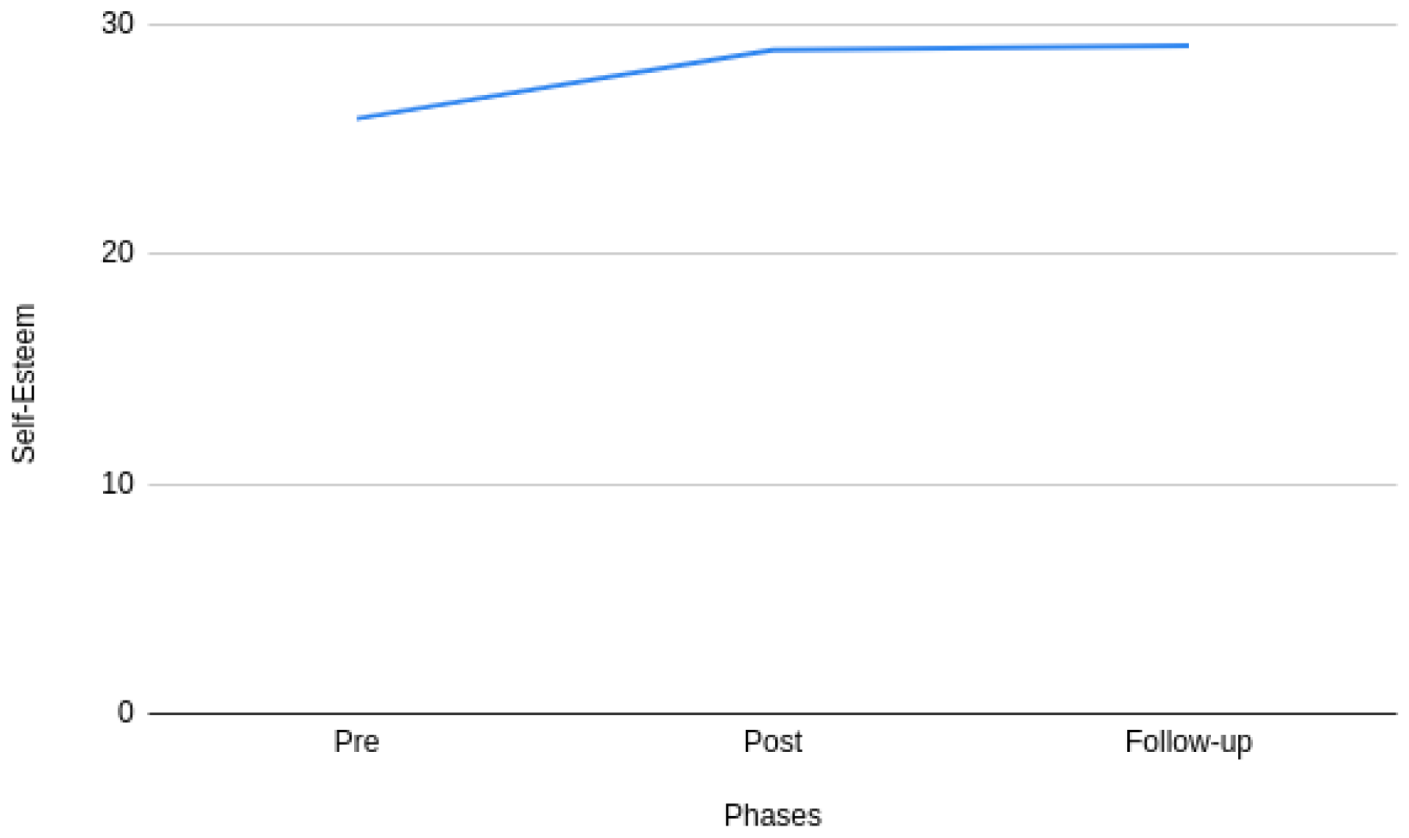
Pre-, post-, and follow-up phases of self-esteem.

Figure 2 and Figure 3 show the changes in the mean stress and self-esteem scores across the pre-, post-, and follow-up phases of the intervention. It can be observed from Figure 2 that there is a significant difference in stress during pre- and post- phases of the intervention. However, it can also be observed that the difference tends to decrease between the post- and follow-up phases of the intervention. A similar pattern can also be observed with self-esteem in Figure 3. More specifically, the mean value of stress and self-esteem improved throughout the study.

Table 5 shows the Post hoc analysis for the pre-, post-, and follow-up phases of intervention for stress and self-esteem. The mean stress scores of the college students during pre- and post-phases changed with a significant difference, 4.32 *±* 0.75, p = 0.00. Pre- and follow-up phases also significantly differed, 4.53 *±* 0.75, p = 0.00. There was no statistically significant difference between the follow-up and post-phases, 0.21 *±* 0.75, p = 1.00. This analysis showed a significant decline in the stress scores of the pre- and post-phases because of the intervention. Self-esteem mean score pre- and post-phases (2.97 *±* 0.75, p = 0.00.) and pre- and follow-up phases (3.17 *±* 0.75, p = 0.00.) were statistically significant. Though there was an increase in the mean score of the post- and follow-up phases, the post hoc analysis revealed that (0.18 *±* 0.75, p = 0.00.) were not statistically significant. Thus, we can conclude that the mindfulness intervention raised the self-esteem scores of the college students involved in placements.

**Table 5.** Post hoc analysis for the pre-, post-, and follow-up phases of intervention for stress and self-esteem.

Variables	Phases		Mean differences (I-J)	Standard error
Stress	Pre-	Post-	4.32 *	0.75
		Follow-up	4.53 *	0.75
	Post-	Pr-	4.32 *	0.75
		Follow-up	0.21	0.75
	Follow-up	Pre-	4.53 *	0.75
		Post-	0.21	0.75
Self-esteem	Pre-	Post-	2.97 *	0.75
		Follow-up	3.17 *	0.75
	Post-	Pre-	2.98 *	0.75
		Follow-up	0.18	0.75
	Follow-up	Pre-	3.17 *	0.75
		Post-	0.18	0.75

* Significance at 0.05 level

## Discussion

This interventional study commenced with a focused group discussion, approved by the institutional ethical review board, using an online platform. Google docs helped collect the data from a hundred college students involved in the placements, and eighty out of a hundred had high stress and low self-esteem. Among eighty, seventy-five benefited from the mindfulness-based intervention. The study’s findings confirmed the existence of high stress and low self-esteem and the impact of mindfulness-based interventions in handling them. Although the post hoc analysis did not reveal a statistically significant change in reducing the participants’ stress, the enhancement of self-esteem and reduction of stress were implied by the mean scores.

The after-effects of COVID-19 on humans are fathomable, and people are now mitigating the negatives and focusing on development and improving their lives. However, college students who need to start new endeavours in the arena of life might feel stagnant because of the added stress of COVID-19 during placements. Furthermore, the consistent stress due to placements and COVID-19 might make the students doubt their ability. It was found in this experimental study that mindfulness-based intervention could serve as a support system throughout these challenging situations. Similarly, Coffey *et al*. ^53^ showed that mindfulness-based intervention improves individuals’ mental health.

On the other hand, self-esteem is a personalised evaluation of one’s confidence. It serves as a powerful tool capable of estimating specific outcomes such as academic success ^46^, happiness in relationships ^54^, and much more. Guidetti *et al*. ^55^ found that mindfulness as a trait mitigates stress levels and enhances meaning in a person’s career. This serves as a protective mechanism against burnout.

Mindfulness has the potential to serve as a protective mechanism throughout a college student’s life, and its impact need not be limited to scenarios such as placements and COVID-19. Buddhism and other spiritual practices across the globe suggest mindfulness as a way of living. These various ideologies found in spiritual practices include the main elements of non-discrimination of psychological distress and positive emotional experiences ^56^. This ideology thereby reduces the partiality to contemplate these kinds of experiences as highs and lows and instead considers acceptance of all human experience as a whole. It was proposed in 47 that the lifelong learning concept will become practical and applicable if the under-estimated domain of learning is placed at the core of the whole structure. For the above scenario to happen, mindfulness has to be incorporated into the curriculum.

### Implications

College students need to take up new roles and make tough decisions in life. In addition, they have to meet the demands, pressure, and expectations from society and the after effects of the pandemic. Thus, mindfulness-based interventions will aid their focus and set a path for a bright future. Frontline workers like psychologists, psychiatric nurses, and psychiatric social workers can teach mindfulness to college students. The awareness and impact of mindfulness-based interventions should be made mandatory to all colleges and universities. In addition, the government could allocate funds and grants to researchers and trainers who conduct research and training programs on mindfulness.

### Limitations and recommendations

Since it is a quasi-experimental study where confounding variables can arise, researchers recommend that future researchers may conduct research with different control groups to describe the influence of independent variables on dependent variables clearly. Purposive sampling was used to conduct this research in a single university. Future research should cover a diverse population, including more colleges in different districts/counties. The same experiment could also be conducted for alumni who are yet to find employment. Focusing on a diverse population and including parameters covering different areas of psychological health will open up new scope for research. Although the post hoc analysis revealed statistically significant results, the mean scores of stress and self-esteem did not continue in the follow-up phase. This could be rectified by assisting the participants to engage more in mindfulness meditation interim sessions. In addition, mindfulness meditation could be introduced as a life skill by creating an online presence on platforms like websites, apps, and courses. This will encourage students to habituate mindfulness meditation as a lifelong learning process.

## Conclusions

The COVID-19 pandemic has made us reflect on all our endeavours. College students devote three-quarters of their time to educational purposes until they graduate and are expected by society to find a suitable placement. Although COVID-19 has impacted and challenged college students’ placements, the psychological problems it has caused can be handled using mindfulness-based intervention. It can also serve as an efficient tool that benefits students preparing for placements, job fairs, or interviews by making them focus on the task at hand and mitigating the impact of COVID-19.

## Consent

Written informed consent for publication of the participants’ details was obtained from the participants.

## Data Availability

Figshare: Underlying data for ’Does online mindfulness-based intervention help college students succeed in their job search during the COVID-19 pandemic?’ https://doi.org/10.6084/m9.figshare.19360955.v4 ^50^ This project contains the following underlying data: Data file: Complete data containing Socio-Economic Scale information for each participant.xlsx Figshare: Extended data for ’Does online mindfulness-based intervention help college students succeed in their job search during the COVID-19 pandemic?’ https://doi.org/10.6084/m9.figshare.19360955.v4 ^50^ This project contains the following extended data: Questionnaire: Demographic questions.docx Data are available under the terms of the Creative Commons Attribution 4.0 International license (CC-BY 4.0).
