# Supplementary material for: Job Search during COVID-19: How Online Mindfulness Intervention helped to Reduce Stress and Enhance the Self-esteem of the Job Seeker
Source: F1000Res. 2022 Nov 4;11:955. Originally published 2022 Aug 18. [Version 2] doi: 10.12688/f1000research.109523.2 (PMC9732500; doi:10.12688/f1000research.109523.2)
Supplement: Peer review history

# Peer review: Reviewer response for version 2

**DOI:** 10.5256/f1000research.139643.r157595

**Published:** 2022-12-08

**Authors:** Nilanga Abeysinghe

**Affiliations:** 1 Sri Lanka Institute of Information Technology, Malabe, Sri Lanka

**Introduction**

 The study consists of a detailed introduction with support from existing literature. It is detailed and sufficiently emphasizes the importance of this study. 

 The authors clearly state the study objective and the hypothesis. However, it would have been useful to have the null hypothesis also stated clearly. 

**Methods**

 The study design is clearly stated.

 The authors have provided sufficient details on the participants and the procedure with enough details for anyone trying to replicate the study. 

**Results**

 This section consists of the necessary information. 

**Discussion**

 The discussion is also satisfactory as it addresses the results with reference to previous literature. The authors have also considered the limitations when drawing conclusions from the results section.

Is the work clearly and accurately presented and does it cite the current literature?

Yes

If applicable, is the statistical analysis and its interpretation appropriate?

Yes

Are all the source data underlying the results available to ensure full reproducibility?

Yes

Is the study design appropriate and is the work technically sound?

Yes

Are the conclusions drawn adequately supported by the results?

Yes

Are sufficient details of methods and analysis provided to allow replication by others?

Yes

Reviewer Expertise:

Counselling psychology

I confirm that I have read this submission and believe that I have an appropriate level of expertise to confirm that it is of an acceptable scientific standard.

# Peer review: Reviewer response for version 2

**DOI:** 10.5256/f1000research.139643.r155041

**Published:** 2022-11-07

**Authors:** Nanchatsan Sakunpong

**Affiliations:** 1 Behaviour Science Research Institute, Srinakharinwirot University, Bangkok, Thailand

I think the authors can address almost all of my comments. However, I suggest to rewrite the Background part to show the gap of knowledge concisely and clearly.

Is the work clearly and accurately presented and does it cite the current literature?

Yes

If applicable, is the statistical analysis and its interpretation appropriate?

Yes

Are all the source data underlying the results available to ensure full reproducibility?

Partly

Is the study design appropriate and is the work technically sound?

Yes

Are the conclusions drawn adequately supported by the results?

Yes

Are sufficient details of methods and analysis provided to allow replication by others?

Partly

Reviewer Expertise:

NA

I confirm that I have read this submission and believe that I have an appropriate level of expertise to confirm that it is of an acceptable scientific standard.

# Response

**Published:** 2022-11-07

**Authors:** Rajalakshmi S.A.

**Affiliations:** Amrita College of Engineering, Amrita Vishwa Vidhyapeetham, Coimbatore, India

Thank you for your time and inputs. Those helped us to improve the quality of the article.

# Peer review: Reviewer response for version 1

**DOI:** 10.5256/f1000research.121032.r148093

**Published:** 2022-09-08

**Authors:** Nanchatsan Sakunpong, Passakorn Koomsiri

**Affiliations:** 1 Behaviour Science Research Institute, Srinakharinwirot University, Bangkok, Thailand; 2 Srinakharinwirot University, Bangkok, Thailand

This study is interesting in how to apply mindfulness with an online intervention which might be appropriate for the digital technology era. However, this study still has some points that can be developed and need to be explained with the following details:

1. The title of the study told us that the program would help in job search success, which was inconsistent with the variables studied by the researcher. Therefore, perhaps changing the title by adding the dependent variable of this research instead of succeed in job search would be more appropriate.
2. In the introduction section, it may be necessary to explain the knowledge gap and how this research can help fill the missing knowledge gap.
3. This study seems to use a quasi-experimental research design but was not specified in the research design section. Instead, it was written to be focus group discussion, which was inconsistent with the actual research methodology used in the study. 
4. Because this study wants to present a new intervention. So, it's important to write a detailed description of each activity (e.g. eating meditation, body scan, mountain meditation - short how-to) that will be helpful to anyone interested in implementing or re-testing the program in the future.
5. Should add the effect size of the program in the results section.
6. Because this study appears to be a quasi-experimental study where confounding variables can arise. So, the recommendation for research in the future can be conducted with additional control groups to be more clearly describe the influence of independent variables on dependent variables. 

Is the work clearly and accurately presented and does it cite the current literature?

Yes

If applicable, is the statistical analysis and its interpretation appropriate?

Yes

Are all the source data underlying the results available to ensure full reproducibility?

Partly

Is the study design appropriate and is the work technically sound?

Yes

Are the conclusions drawn adequately supported by the results?

Yes

Are sufficient details of methods and analysis provided to allow replication by others?

Partly

Reviewer Expertise:

Positive psychology, Psychology of gender and sexual orientation

We confirm that we have read this submission and believe that we have an appropriate level of expertise to confirm that it is of an acceptable scientific standard, however we have significant reservations, as outlined above.

# Response

**Published:** 2022-10-08

**Authors:** Rajalakshmi S.A.

**Affiliations:** Amrita College of Engineering, Amrita Vishwa Vidhyapeetham, Coimbatore, India

We are happy to see your review of our paper, and we are grateful for your time and suggestions. We have incorporated your comments into our work, and please continue to guide and support us.
